# Mapping VEXAS‐associated and rare *UBA1* variants in the United Kingdom: Insights from patient cohorts and the general population

**DOI:** 10.1111/bjh.20176

**Published:** 2025-05-25

**Authors:** Ana Martinez Rodriguez, Leon Chang, Dorota Rowczenio, Alexandra Smith, Ebun Omoyinmi, James A. Poulter, Andrew McGregor, Sebastian Francis, Roochi Trikha, Adam Al‐Hakim, Kar Lok Kong, David G. Kent, Helen Lachmann, Austin Kulasekararaj, Catherine Cargo, Sinisa Savic

**Affiliations:** ^1^ Leeds Institute of Rheumatic and Musculoskeletal Medicine University of Leeds Leeds UK; ^2^ National Amyloidosis Centre London UK; ^3^ Epidemiology and Cancer Statistics Group, Department of Health Sciences University of York York UK; ^4^ Leeds Institute of Medical Research University of Leeds Leeds UK; ^5^ Department of Haematology Newcastle upon Tyne Hospitals NHS Foundation Trust Newcastle upon Tyne UK; ^6^ Department of Haematology Sheffield Teaching Hospitals NHS Trust Sheffield UK; ^7^ Haematology Department King's College Hospital NHS Foundation Trust London UK; ^8^ Department of Clinical Immunology and Allergy St James's University Hospital Leeds UK; ^9^ Synnovis, Laboratory for Molecular Haemato‐Oncology King's College Hospital NHS Foundation Trust London UK; ^10^ Department of Biology, Centre for Blood Research York Biomedical Research Institute University of York York UK; ^11^ Haematological Malignancy Diagnostic Service, St James's University Hospital Leeds UK; ^12^ NIHR Leeds Biomedical Research Centre Leeds UK

**Keywords:** MDS, UBA1, VEXAS

## Abstract

Somatic mutations in *UBA1* are linked to VEXAS syndrome, a late‐onset inflammatory disorder with rheumatological and haematological features, primarily affecting elderly men. This study examines the epidemiology of VEXAS in the United Kingdom using genomic databases and patient cohorts to estimate prevalence, identify novel *UBA1* variants and predict their pathogenicity. Analysing data from the UK Biobank, 100 000 Genomes Project and clinical diagnostic laboratories, we found that VEXAS prevalence in UK males over 50 is lower than in US‐based cohorts. Notably, canonical (Met41) *UBA1* mutations appear in ~1% of individuals with autoinflammatory disorders who have not been referred to haematology. However, among those investigated for myeloid malignancies, VEXAS is relatively common, with an estimated incidence of 1.51 per 100 000, or 171 new cases each year. We identified 47 *UBA1* non‐Met41 variants of uncertain significance, with several showing clonal dominance and clustering in functional domains, suggesting potential pathogenicity. Clinical presentation associated with non‐Met41 variants often diverged from classical VEXAS features, underscoring the need for further studies. Our findings highlight the importance of broader screening for both canonical and non‐canonical *UBA1* mutations to improve understanding of VEXAS syndrome and its underlying mechanisms.

## Graphical abstract

VEXAS syndrome is a late‐onset inflammatory disorder with rheumatological and haematological features. Epidemiological studies of VEXAS syndrome so far have been limited. Analysis of various UK cohorts estimates the incidence of VEXAS to be 1.51/100 000, or 171 new cases in the population of men over the age of 50 who are being investigated for myeloid malignancies. Forty‐seven non‐Met41 *UBA1* variants of unknown significance were also identified, with several showing clonal dominance and clustering in functional domains, suggesting potential pathogenicity. Clinical presentation associated with non‐Met41 variants often diverged from classical VEXAS features, underscoring the need for further studies.

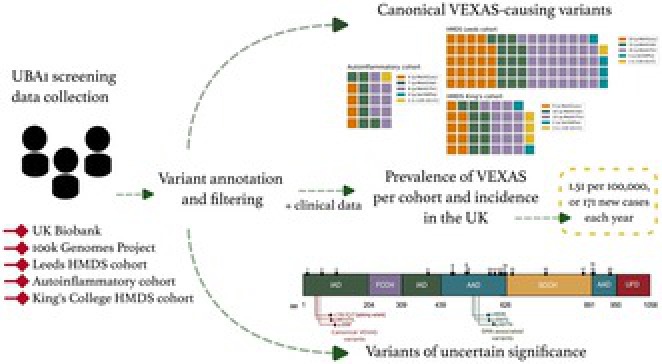

## INTRODUCTION

The *ubiquitin‐like modifier‐activating enzyme 1* (*UBA1*) gene encodes the major E1‐activating enzyme, essential for initiating ubiquitylation—a critical process for maintaining intracellular homeostasis. Until recently, the only known disease association illustrating the enzyme's essential nature was with X‐linked spinal muscular atrophy (SMA) (MIM #301830), a condition characterized by severe neonatal‐onset hypotonia, multiple congenital contractures and infantile death.^1^ SMA‐related variants were postulated to result in partial reduction of the protein expression or impaired interaction of UBA1 with an intracellular complex that is important for maintaining neuronal cell viability.^1^ A recent study has shown that these variants cause mild to moderate temperature‐dependent reduction in UBA1 enzyme activity.^2^ Subsequently, somatic (acquired) mutations in *UBA1* have been linked to a late‐onset inflammatory disorder known as VEXAS (vacuoles, E1 enzyme, X‐linked, autoinflammatory, somatic) syndrome.^3^ VEXAS syndrome (MIM #301054) is a complex condition, presenting with overlapping rheumatological and haematological features, including relapsing polychondritis, neutrophilic dermatosis, macrocytic anaemia and concomitant myelodysplastic syndrome (MDS) in up to 50% of patients.^4^, ^5^

In the original description of VEXAS syndrome, all pathogenic mutations were located at the p.Met41 hotspot, which disrupts the translation of the cytoplasmic isoform UBA1b and leads to the expression of the catalytically impaired UBA1c isoform.^3^ Subsequently, additional splice‐acceptor variants have been reported at the 5'‐intron/exon boundary of exon 3 (c.118‐1G>C and c.118‐2A>C), which produce clinical endotypes similar to the canonical p.Met41 variants.^6^ Pathogenic variants outside the p.Met41 hotspot have since been discovered, which do not affect UBA1 isoform expression but instead modify the protein's catalytic activity.^2^, ^6^, ^7^ Additionally, numerous rare, uncharacterized variants of uncertain significance (VUS) have been identified through large‐scale genomic studies of related conditions, such as MDS.^8^, ^9^

To date, only a few studies have examined the prevalence and incidence of VEXAS syndrome in the general population. One such study, conducted in the United States, estimated a prevalence of 1 in 4269 for males over 50 years of age and 1 in 26 238 for females over 50 years of age.^10^ The higher prevalence in males aligns with the original description of the condition, as with subsequent studies, which have shown that VEXAS predominantly affects males due to *UBA1* being on the X chromosome.

To provide a more comprehensive understanding of rare *UBA1* variants and the prevalence of VEXAS syndrome across general and patient‐based populations, we leveraged data from existing UK‐based general population cohorts and a harmonized healthcare system. We determined the frequency of canonical and non‐canonical *UBA1* variants and estimated the prevalence of VEXAS syndrome within these populations, and in overlapping conditions such as MDS and related myeloid disorders. Additionally, we annotated the identified VUSs with pathogenicity predictions and explored their implications with the disease.

## METHODS

### Genomic databases

We had access to data from two UK‐based genomic databases: The UK Biobank and the 100 000 Genome Project (100kGP). Further information about these databases and analysis pipelines can be found under Supplementary Methods.

### Data from routine laboratory testing

Considering the heterogeneous clinical presentation of VEXAS syndrome, *UBA1* genetic testing in the United Kingdom is available in multiple diagnostic laboratories. It is included in standard gene panels for investigating unexplained cytopenias, suspected myeloid disorders and autoinflammatory conditions. For this study, we accessed genetic sequencing data from two accredited Haematological Malignancy Diagnostic Services (HMDS) laboratories for cytopenia and myeloid disorder investigations and a national reference laboratory for autoinflammatory syndromes.

Leeds HMDS was the first centre in the United Kingdom to include *UBA1* testing after VEXAS was identified, providing the longest experience in detecting both pathogenic and novel variants. Additionally, Leeds HMDS identifies patients for inclusion in the Haematological Malignancy Research Network (HMRN), the United Kingdom's largest database on haematological malignancies. Established in 2004, HMRN delivers population‐based research on incidence, treatment and survival.^11^ We used combined data from Leeds HMDS and HMRN to estimate the prevalence of VEXAS syndrome across the United Kingdom. The second HMDS laboratory based in King's College Hospital, London, covering the south‐east of the country, serves a different geographic area but provides similar testing services and methodologies for *UBA1* detection. Lastly, the Royal Free London NHS Foundation Trust offers genetic screening for autoinflammatory conditions with a panel that includes systemic autoinflammatory disease (SAID) genes. Laboratories' gene panels (Table S1) and sequencing details can be found in Supplementary Methods. UBA1 sequencing coverage and limit of detection for each dataset are provided in Table S2.

## RESULTS

### Prevalence of canonical *UBA1* variants across different cohorts

To estimate the prevalence of VEXAS syndrome in the UK population, we interrogated two genomic databases and three genetic screening centres to identify cases with canonical *UBA1* variants affecting the p.Met41 residue. These variants on the X chromosome have previously been shown to exhibit nearly 100% disease penetrance in males; however, their pathogenic potential is less clear when present in the heterozygous state in women.

In the UK Biobank, which includes exome sequencing data from 469 617 individuals, we identified only a single case with a canonical *UBA1* mutation. This was a male over 50 years of age, harbouring the known VEXAS‐causing *UBA1* splice mutation c.118‐1G>C. Clinical manifestations consistent with VEXAS were noted as part of the phenotype at the time of enrolment. Based on this dataset, the estimated prevalence of VEXAS syndrome is 1 in 469 617 (95% CI 1:84 287–1:18 548 881). When adjusted for age and sex to focus on males of 50 years of age and over—where the condition is most frequently reported—the prevalence increases to 1 in 165 190 (95% CI 1:29 648–1:6 524 656). Interrogation of the 100kGP dataset, which includes whole genomes of participants recruited for either rare diseases or cancer, similarly identified only one case with the known pathogenic *UBA1* variant c.121A>C, p.Met41Leu (variant allele frequency [VAF]: 8%), associated with VEXAS syndrome. The identified case was a male over 50 years of age in the haematological malignancy cohort (HAEMONC) consisting of 842 cases (95% CI 1:151–1:33 257). More specifically, this case was found among 77 individuals recruited with myeloproliferative neoplasms. No cases were identified in the rare disease cohort (0 in 62 729 individuals) or among other non‐haematological malignancies.

### Prevalence of canonical *UBA1* variants identified by diagnostic laboratories

**FIGURE 1:**
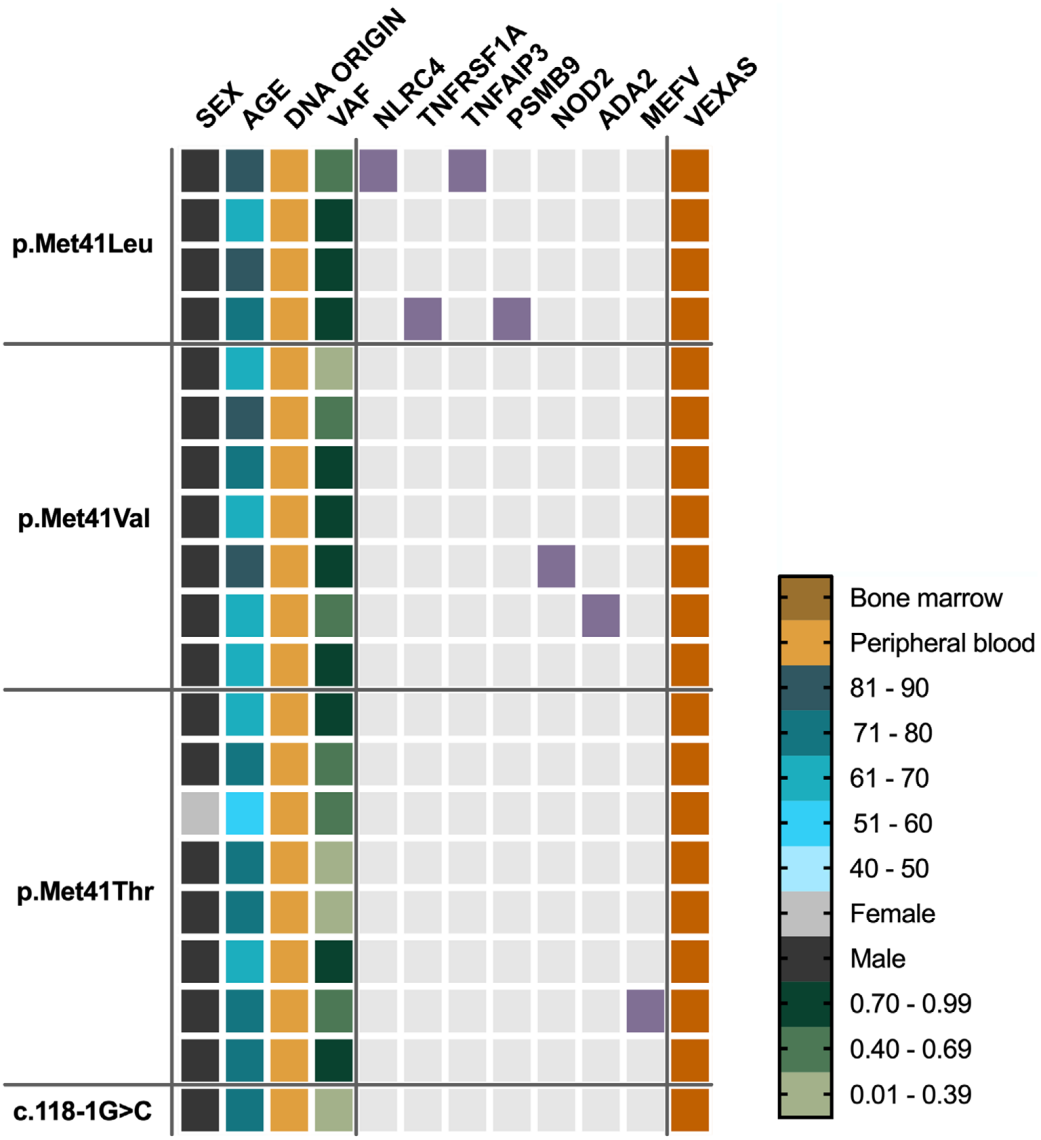
Summary of cases in the autoinflammatory cohort. Twenty individuals from the autoinflammatory cohort carried canonical pathogenic *UBA1* variants associated with VEXAS. Age, sex, DNA sample origin, diagnoses and *UBA1* variant allele fractions (VAFs) are presented for each case. Cases clinically diagnosed with VEXAS are highlighted in orange. Individuals carrying co‐mutations in genes associated with systemic autoinflammatory disease (SAID) are highlighted in purple. The mutational origins of these co‐mutations have not been confirmed. All the co‐mutations were reported as VUSs.

We reviewed data from 2769 cases screened for autoinflammatory disorders at the reference laboratory of the Royal Free London NHS Foundation Trust (screened from July 2020 to June 2024). Of 63 referrals for suspected VEXAS syndrome, 16 (25%) were found to carry pathogenic mutations. Four additional cases were identified among the remaining cohort, totalling 20 diagnoses (19 male, one female). (Figure 1; Figure S1). The most common mutations were p.Met41Thr (40%) and p.Met41Val (35%). The estimated prevalence of VEXAS in suspected autoinflammatory cases was 1 in 138 (95% CI 1:90–1:227), rising to 1 in 16 for males over 50 (95% CI 1:10–1:26) and 1 in 260 for females over 50 (95% CI 1:46–1: 10 269). All co‐mutations in SAID genes were reported as VUSs.

**FIGURE 2:**
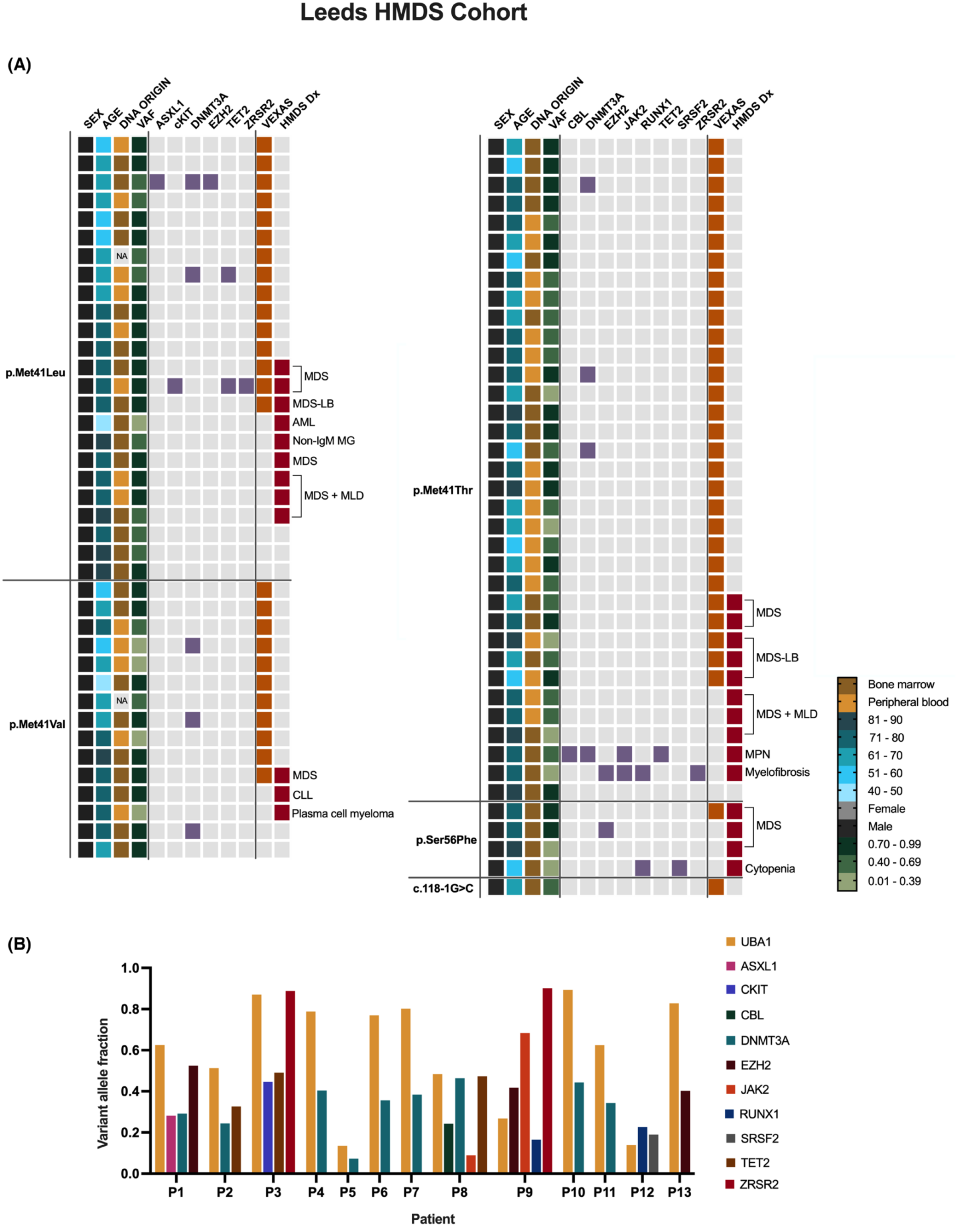
Summary of cases in the HMDS Leeds cohort. (A) Cases carrying canonical pathogenic VEXAS‐causing *UBA1* variants. Age, sex, DNA sample origin, diagnoses and *UBA1* variant allele fractions (VAFs) are displayed for each case; alongside co‐mutations in genes within the HMDS virtual gene panel (highlighted in purple). Cases clinically diagnosed with VEXAS are highlighted in orange. HMDS diagnoses (Red) abbreviations: AML, acute myeloid anaemia; CLL, chronic lymphocytic leukaemia; LB, low blasts; MDS, Myelodysplastic syndrome; MLD, multilineage dysplasia; MPN, myeloproliferative neoplasm; non‐IgM MG, non‐IgM monoclonal gammopathy. (B) 13 patients with canonical pathogenic VEXAS‐causing *UBA1* variants with confirmed pathogenic co‐mutations in genes associated with haematological malignancies. VAFs for each mutation are shown across the 13 patients (P). Variants in *DNMT3A* were the most frequently observed in mutated *UBA1* cases. The mutational origins of these co‐mutations have not been confirmed, and all were reported as pathogenic. NA denotes missing data.

Further analysis was conducted on patient cohorts investigated for unexplained cytopenias and suspected myeloid malignancies using data from Leeds Teaching Hospitals NHS Trust and King's College Hospital. In the Leeds HMDS cohort, 9990 individuals (5737 men, average age: 64 years, range: newborn to 99) were tested between January 2020 and March 2024. Seventy‐nine males had canonical VEXAS‐causing *UBA1* mutations, most commonly p.Met41Thr (44.3%), followed by p.Met41Leu (30.4%) and p.Met41Val (19.0%) (Figure 2A; Figure S1). VEXAS was confirmed in 55 cases, with an estimated prevalence of 1 in 181 overall (95% CI 1:139–1:241) and 1 in 104 among men over 50 years (95% CI 1:80–1:139).

Among the 55 confirmed VEXAS cases, 26 were suspected at the time of testing, while others were investigated for MDS or unexplained cytopenia. Eleven patients also had lower risk MDS (Figure 2A). Additional diagnoses included acute myeloid leukaemia (AML), multiple myeloma, chronic lymphocytic leukaemia (CLL) and cytopenia of unknown cause. Eight VEXAS patients harboured MDS‐associated mutations, most commonly *DNMT3A* (7/8 cases) (Figure 2A). Of 13 *UBA1* mutation carriers, 10 had predominant *UBA1* clones compared to MDS‐associated co‐mutations (Figure 2B). All these co‐mutations were reported as pathogenic. The VAFs of canonical *UBA1* mutations ranged from 0.13 to 0.92, and no correlation was identified with age at sample collection (Figure S2).

**FIGURE 3:**
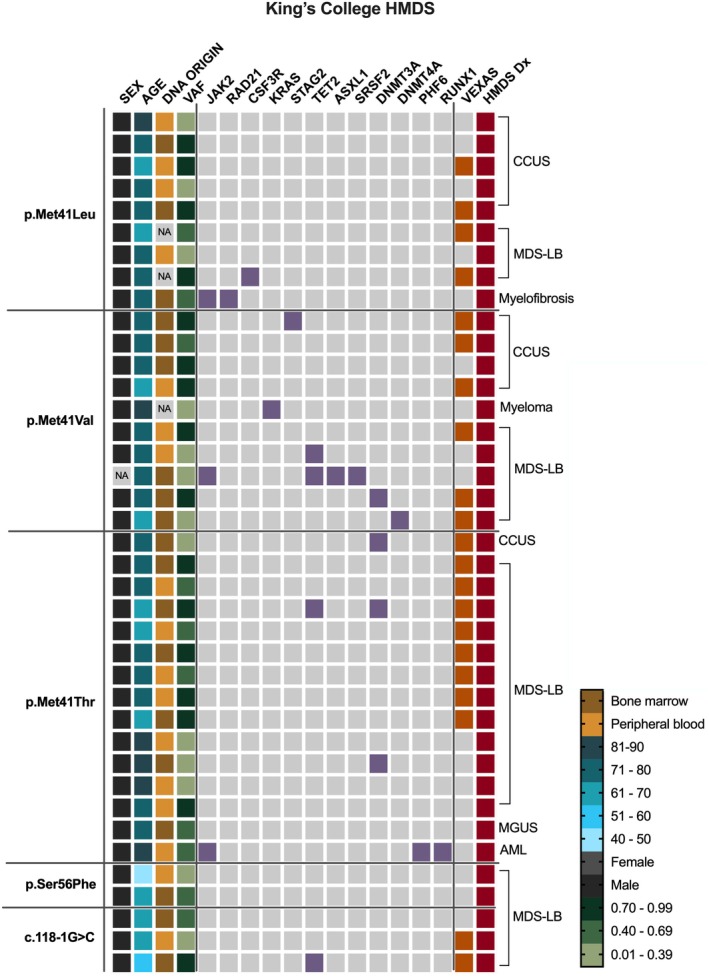
Summary of cases in the HMDS King's cohort. Cases with canonical pathogenic VEXAS‐causing *UBA1* variants. Age, sex, DNA sample origin, diagnoses and *UBA1* variant allele fractions (VAFs) are displayed for each case; alongside co‐mutations in genes within the HMDS virtual gene panel (highlighted in purple). Cases clinically diagnosed with VEXAS are highlighted in orange. HMDS diagnoses (Red) abbreviations: AML, acute myeloid anaemia; CCUS, clonal cytopenia of unknown significance; LB, low blasts; MDS, myelodysplastic syndrome; MGUS, monoclonal gammopathy of undetermined significance. The mutational origins of these co‐mutations have not been confirmed, and all were reported as pathogenic. NA denotes missing data.

The HMDS cohort from King's College Hospital included 5816 individuals tested between November 2022 and October 2024. Among them, 38 males (and one patient of unknown sex) harboured canonical VEXAS‐causing mutations, most commonly p.Met41Thr (38.5%), followed by p.Met41Val (25.6%) and p.Met41Leu (23%) (Figure 3; Figure S1). VEXAS was confirmed in 21 cases, with an estimated prevalence of 1 in 277 (95% CI 1:181–447).

In this cohort, *UBA1* variant testing was primarily conducted for suspected MDS. There were 25 cases of MDS, and of those diagnosed with VEXAS, 14 (66.6%) had a concurrent MDS diagnosis. Among individuals with canonical UBA1 mutations but no confirmed VEXAS, most were diagnosed with MDS (55.5%) or clonal cytopenia of unknown significance (CCUS) (21%) (Figure 3). Overall, 33.3% of VEXAS patients carried additional MDS‐associated mutations. All these co‐mutations were reported as pathogenic.

To investigate whether the size of the *UBA1* clone is determined by the patient's age, we analysed the correlation between VAF and age at the time of sampling of all cases from the diagnostic laboratories. Consistent with findings from the Leeds HMDS cohort, no significant correlation was observed (Figure S3).

### Prevalence of VEXAS syndrome in patients investigated for suspected myeloid malignancies and incidence in the United Kingdom

We combined data from the Leeds and King's HMDS cohorts to assess VEXAS prevalence among patients investigated for myeloid disorders. Among 15 806 patients, 83 were diagnosed with VEXAS, yielding an estimated prevalence of 1 in 190 (0.52%). Using data from the HMRN (Haematological Malignancy Research Network),^12^ we estimated the annual incidence of VEXAS syndrome in the UK population. The incidence rate was calculated using population estimates from the Office of National Statistics for the regions, and the annual number of new cases diagnosed in the United Kingdom was estimated by applying the resulting rate to the UK population. This was calculated to be 0.06 per 100 000. When restricting the analysis to males over 50 years of age, the incidence increased to 1.51 per 100 000, or an estimated 171 new cases each year. A summary of the individual cohorts' prevalences can be found in Table 1.

**TABLE 1:** Summary of estimated prevalences in the five study cohorts.

	Period of screening (months)	Participants/patients screened	VEXAS cases	Prevalence
UK Biobank	39	469 617	1^a^	1 in 469 617 (95% CI 1:84 287–1:18 548 881)
				1 in 165 190 (95% CI 1:29 648–1:6 524 656)^b^
100K Genomes Project	45	842 (HAEMONC)	1^a^	1 in 842 (95% CI 1:151–1:33 257)
				1 in 273 (95% CI: 1:49–1:10 783)^b^
		77 000 (Rare disease)	0	N/A
Autoinflammatory diagnostic cohort	47	2769	20	1 in 138 (95% CI 1:90–1:227)
				1 in 16 (95% CI 1:10–1:26)^b^
HMDS Leeds	51	9990	55	1 in 181 (95% CI 1:139–1:241)
				1 in 109 (95% CI 1:80–1:139)^b^
HMDS King's College	24	5816	21	1 in 277 (95% CI 1:181–447)
				N/A^b^

*Note*: The estimated prevalence from each cohort. Estimations are provided with 95% confidence intervals (CI). The 100K Genomes Project is divided into Cancer and Rare disease subcohorts and only samples from whole blood were included in the analysis. N/A, not available.

^a^ An individual identified with a canonical VEXAS‐causing variant and symptoms in keeping with VEXAS syndrome but not formally diagnosed.

^b^ The prevalence adjusted for males ≥50 years of age.

### Identification of *UBA1* variants of unknown significance (VUS)

Recent studies have identified rare *UBA1* variants outside the p.Met41 position that are associated with VEXAS‐like phenotypes, though these cases often present with milder inflammatory manifestations. Some of these variants have also been reported in patients with MDS, further suggesting that they might have a specific role in disease pathogenesis.^8^, ^9^ To investigate the overall distribution of rare *UBA1* variants and to determine whether any cluster with specific disease phenotypes, we interrogated the same datasets we used to establish the prevalence of the canonical *UBA1* mutations.

From the UK Biobank dataset, we identified 400 coding variants with anticipated functional effects. After filtering out common and unlikely disease‐causing VUSs, 176 heterozygous variants were identified in 379 females. Due to limitations in the bioinformatic pipeline and lack of matched controls, distinguishing somatic from germline variants was not possible. In males, 114 hemizygous variants were found in 185 participants, and two suspected mosaic (genotyped as heterozygous) cases (*UBA1*c.2183G>A, p.Asn728Ser and c.2587A>G, p.Val863Met). These variants, classified as ‘likely benign’ or ‘ambiguous’ by AlphaMissense, were unlikely to be pathogenic, with no consistent clinical phenotype (Table S3). We identified nine other variants which had less than four mutant calls. These were not present in the whole genome sequencing calls of the corresponding participant and, therefore, were assigned as possible artefacts and not included in the study.

Whole genomes from the 100kGP cohorts were analysed for novel pathogenic *UBA1* variants using the same criteria as the UK Biobank analysis. Among HAEMONC tumour samples, five VUSs were identified alongside the previously described pathogenic p.Met41Leu variant, all in AML participants (Table S4). Analysis of male germline genomes across rare disease and cancer programmes identified 58 rare *UBA1* variants in 92 participants; all variants were presented as hemizygous and presumed to be of germline origin and excluded from this study.

**FIGURE 4:**
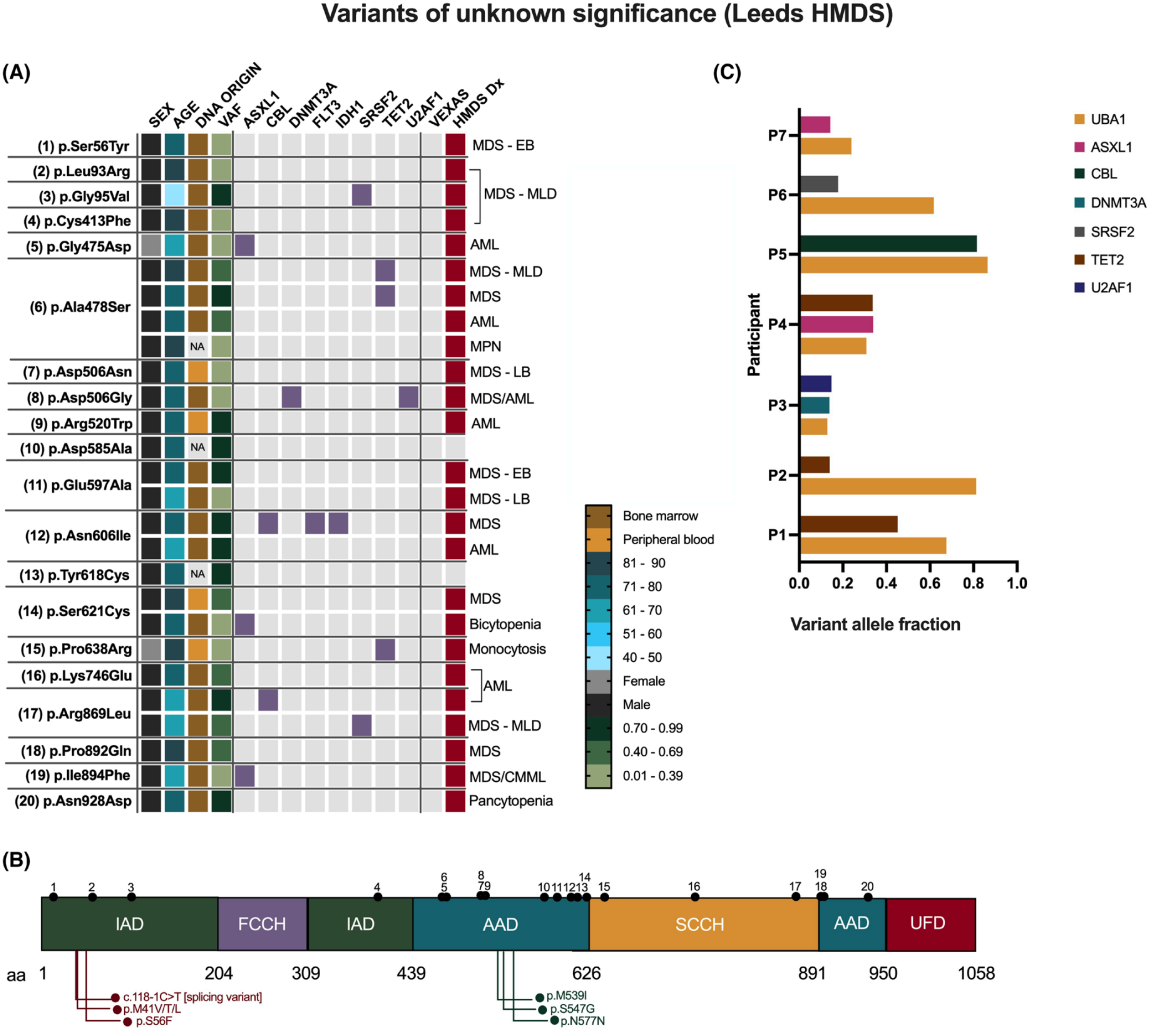
Summary of variants of uncertain significance (VUSs) identified in the HMDS Leeds cohort. (A) Age, sex, DNA sample origin, diagnoses and variant allele fractions (VAFs) of UBA1 VUSs are displayed for each case; alongside co‐mutations in genes within the HMDS virtual gene panel (highlighted in purple). None of the patients received a diagnosis of VEXAS syndrome. HMDS diagnoses (Red) abbreviations: AML, acute myeloid anaemia; CMML, chronic myelomonocytic leukaemia; EB, excess blasts; LB, low blasts; MDS, myelodysplastic syndrome; MLD, multilineage dysplasia; MPN, myeloproliferative neoplasm. (B) VUSs (assigned numbers 1–20; black points) are mapped to their corresponding amino acid (aa) location on the primary structure of UBA1. Validated pathogenic UBA1 variants are annotated with red points and variants associated with X‐linked spinal muscular atrophy as green points. UBA1 domains are as follows: AAD, active adenylation domain; FCCH, first catalytic cysteine half domain; IAD, inactive adenylation domain; SCCH, second catalytic cysteine half domain; UFD, ubiquitin fold domain. (C) VUSs and confirmed pathogenic co‐mutations in genes associated with haematological malignancies. VAFs from these mutations are shown across seven patients. Variants in *TET2* were the most frequently observed in mutated *UBA1* cases. The mutational origins of these co‐mutations have not been confirmed, and all were reported as pathogenic. NA denotes missing data.

Twenty VUSs from the Leeds HMDS cohort were identified using pathogenicity annotation filters (Table 2). These variants, found in 27 patients (25 men), were not associated with VEXAS syndrome. Most patients had MDS or AML and carried additional haematological malignancy‐associated mutations. VAFs ranged from 7.4% to 93.1%, similar to canonical *UBA1* variants. Mapping revealed most variants clustered within the active adenylation domain (AAD), which interacts with ubiquitin, suggesting potential biological impact (Figure 4B). In three of seven cases for which we obtained the VAFs of MDS‐causing variants, the *UBA1* clone was dominant over the other co‐occurring mutations (Figure 4C).

**TABLE 2:** UBA1 variants of uncertain significance (VUSs) in Leeds and King's College Hospital HMDS cohorts.

Leeds HMDS					King's college HMDS				
Variant	gnomAD	CADD score	AM score	REVEL score	Variant	gnomAD	CADD score	AM score	REVEL score
c.167C>A; p.(Ser56Tyr)	NA	25.5	0.988	0.665	c.20C>T; p.(Ser7Phe)	8.26E‐07	25.1	0.6283	0.275
c.278 T>G; p.(Leu93Arg)	NA	26.8	0.9931	0.682	c.130A>G; p.(Asn44Asp)	NA	23.8	0.255	0.105
c.284G>T; p.(Gly95Val)	NA	26	0.9938	0.958	c.250G>T; p.(Gly84Cys)	NA	24.8	0.9654	0.957
c.1238G>T; p.(Cys413Phe)	NA	26.1	0.6785	0.455	c.338C>G; p.(Ser113Cys)	5.77E‐06	24	0.2163	0.277
c.1424G>A; p.(Gly475Asp)	NA	27.1	0.9992	0.902	c.370G>A; p.(Gly124Ser)	9.09E‐06	24.1	0.1641	0.561
c.1432G>T; p.(Ala478Ser)	NA	26.6	0.7756	0.598	c.406G>A; p.(Ala136Thr)	9.92E‐06	24.1	0.1711	0.169
c.1516G>A; p.(Asp506Asn)	NA	28.6	0.9989	0.821	c.415A>C; p.(Asn139His)	NA	24.4	0.9688	0.737
c.1517A>G; p.(Asp506Gly)	NA	28.7	0.9994	0.902	c.458A>G; p.(Glu153Gly)	2.48E‐06	24.7	0.1076	0.311
c.1558C>T; p.(Arg520Trp)	NA	26.5	0.8919	0.523	c.575G>A; p.(Arg192Gln)	2.17E‐05	22.3	0.1001	0.213
c.1754A>C; p.(Asp585Ala)	NA	27.2	0.9973	0.545	c.901A>G; p.(Ile301Val)	NA	27.6	0.0756	0.082
c.1790A>C; p.(Glu597Ala)	NA	27.6	0.9871	0.631	c.1043G>C; p.(Arg348Pro)	8.33E‐07	21.9	0.4076	0.112
c.1817A>T; p.(Asn606Ile)	NA	27.7	0.9505	0.506	c.1064C>T; p.(Ala355Val)	3.30E‐06	24.5	0.2862	0.164
c.1853A>G; p.(Tyr618Cys)	NA	27	0.9901	0.508	c.1339_1340delinsTA; p.(Arg447Tyr)	NA	—	—	—
c.1861A>T; p.(Ser621Cys)	NA	23.6	0.708	0.51	c.1400G>A; p.(Gly467Asp)	2.81E‐05	24	0.3696	0.089
c.1913C>G; p.(Pro638Arg)	NA	26.3	0.9994	0.937	c.1418 T>C; p.(Leu473Pro)	NA	27.5	0.9906	0.649
c.2236A>G; p.(Lys746Glu)	NA	26.4	0.9993	0.836	c.1432G>T; p.(Ala478Ser)	NA	26.6	0.7756	0.598
c.2606G>T; p.(Arg869Leu)	NA	29.6	0.9909	0.797	c.1486G>A; p.(Glu496Lys)	5.59E‐04	21.4	0.0748	0.102
c. 2675C>A; p.(Pro892Gln)	NA	26.1	0.9965	0.922	c.1561C>T; p.(Pro521Ser)	8.26E‐07	23.5	0.1896	0.178
c.2680A>T; p.(Ile894Phe)	NA	25.8	0.9682	0.658	c.1650C>G; p.(Asn550Lys)	NA	22.1	0.8806	0.268
c.2782A>G; p.(Asn928Asp)	NA	25.8	0.9108	0.365	c.1853A>G; p.(Tyr618Cys)	NA	24.8	0.9901	0.508
					c.1861A>T; p.(Ser621Cys)	NA	23.6	0.708	0.51
					c.2078G>A; p.(Arg693His)	1.82E‐05	22.7	0.0738	0.122
					c.2308A>C; p.(Asn770His)	1.20E‐03	23.6	0.2906	0.608
					c.2309A>G; p.(Asn770Ser)	4.13E‐05	23.3	0.1432	0.603
					c.2627C>T; p.(Pro876Leu)	NA	24.9	0.1576	0.141
					c.2668A>T; p.(Ile890Phe)	NA	25	0.9975	0.743
					c.2827C>G; p.(Pro943Ala)	NA	21.6	0.0936	0.17
					c.2941‐17_2941‐14delinsCCCT; p.?	NA	—	—	—
					c.3041C>G; p.(Pro1014Arg)	NA	22.9	0.1333	0.134
					c.3095G>A; p.(Arg1032Gln)	1.49E‐05	22.6	0.098	0.086

*Note*: Pathogenicity annotation for the variants of unknown significance (VUS) identified in *UBA1* (NM_003334.4). These VUS have been filtered on CADD ≥ 20 and gnomAD MAF < 0.01 (1%). AlphaMissense (AM) and Rare Exome Variant Ensemble Learner (REVEL) scores are provided for Supporting Information. Recommended thresholds for pathogenicity: CADD score ≥ 20; AM score, 0.56–1.00 = likely pathogenic, 0.34–0.56 = ambiguous; REVEL score > 0.7. Variants found in both cohorts are highlighted in grey. Population allele frequencies retrieved from gnomAD v4.1.0.

Abbreviation: HMDS, Haematological Malignancy Diagnostic Services.

**FIGURE 5:**
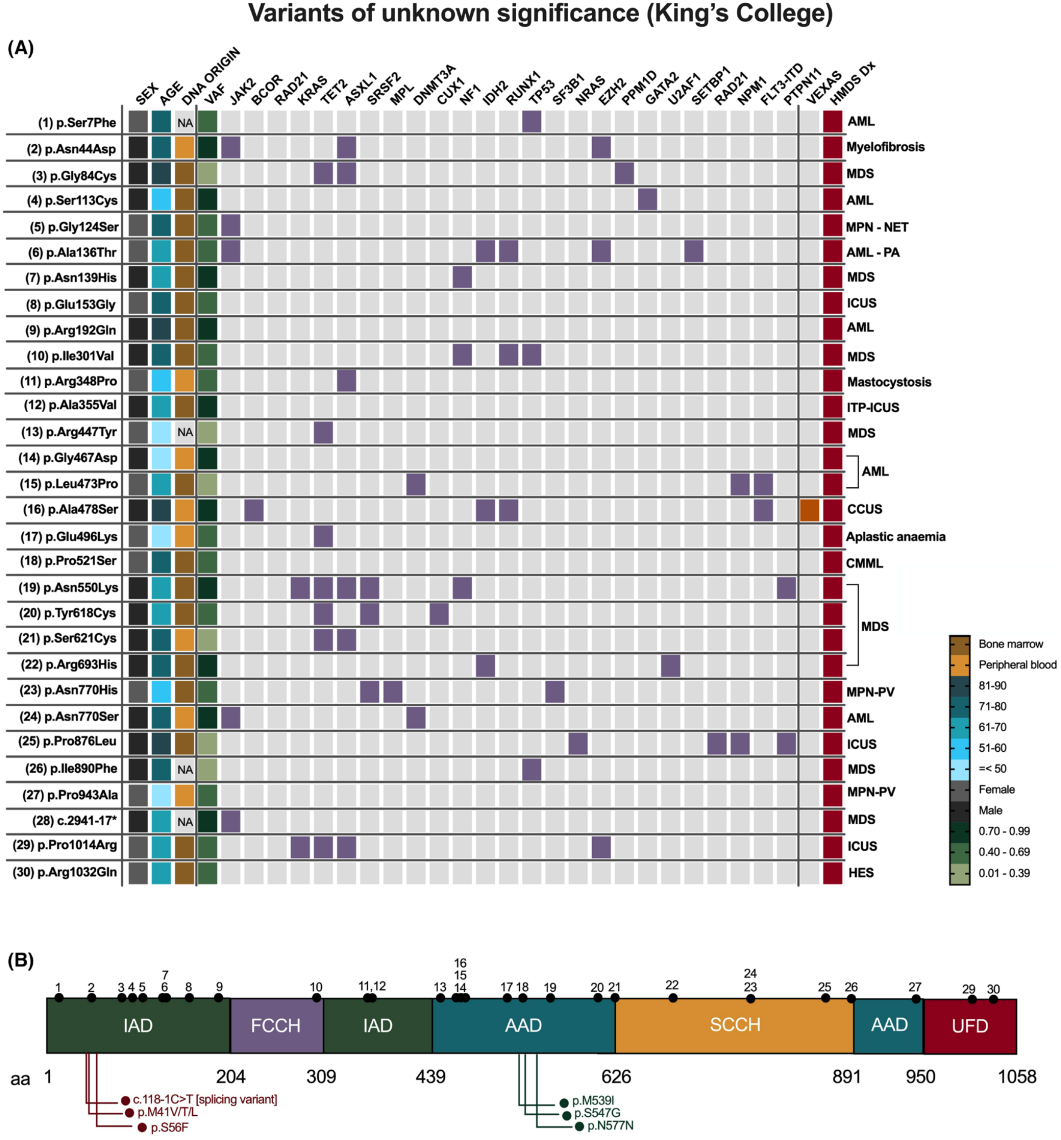
Summary of variants of uncertain significance (VUSs) identified in the HMDS King's College cohort. (A) Age, sex, DNA sample origin, diagnoses and variant allele fractions (VAFs) of UBA1 VUSs are displayed for each case; alongside co‐mutations in genes within the HMDS virtual gene panel (highlighted in purple). Cases clinically diagnosed with VEXAS are highlighted in orange. HMDS diagnoses (red) abbreviations: AML, acute myeloid anaemia; CCUS, clonal cytopenia of unknown significance; CMML, chronic myelomonocytic leukaemia; HES, hypereosinophilic syndrome; ICUS, idiopathic cytopenia of unknown significance; MDS, myelodysplastic syndrome; MPN, myeloproliferative neoplasm. (B) VUSs (assigned numbers 1–30; black points) are mapped to their corresponding amino acid (aa) location on the primary structure of UBA1. Validated pathogenic UBA1 variants are annotated with red points and variants associated with X‐linked spinal muscular atrophy as green points. UBA1 domains are as follows: AAD, active adenylation domain; FCCH, first catalytic cysteine half domain; IAD, inactive adenylation domain; SCCH, second catalytic cysteine half domain; UFD, ubiquitin fold domain. * c.2941‐17_2941‐14delinsCCCT. The mutational origins of these co‐mutations have not been confirmed and all were reported as pathogenic. NA denotes missing data.

In the HMDS King's College cohort, we identified 30 pathogenic‐annotated VUSs (Table 2). Three of these were also detected in the HMDS Leeds cohort: p.Ala478Ser, p.Tyr618Cys and p.Ser621Cys. The first and last have been reported previously.^7^, ^8^, ^10^ The carrier of p.Ala478Ser was the only patient diagnosed with VEXAS (Figure 5A). These variants were distributed across the whole extension of the primary structure of the protein (Figure 5B).

## DISCUSSION

Our study aimed to provide novel insights into the epidemiology of VEXAS syndrome in the United Kingdom, estimate its prevalence in related disorders and assess the pathogenic potential of rare *UBA1* variants using genomic databases and patient cohorts.

Our analysis of UK Biobank data suggests a significantly lower prevalence of VEXAS syndrome in the UK than previously reported in other countries.^10^, ^13^ We identified only one canonical *UBA1* mutation, yielding a potential case prevalence of 1 in 165 190 for men over 50, compared to 1 in 4269 reported in a US regional health system.^10^ Based on this, we would have expected 41 cases in the UK Biobank. This discrepancy could be attributed to differences in study populations, recruitment criteria and sequencing methodologies. Unlike the UK Biobank, whose participants were recruited from the general population, participants in the US Geisinger cohort, which reported the highest prevalence of VEXAS syndrome, were individuals who sought healthcare at a Geisinger facility, suggesting that this cohort may have had a higher overall disease burden. Furthermore, the UK Biobank's age range (40–69 years) may underrepresent the older demographic most affected by VEXAS, and given the limitations in sequencing depth, low VAF *UBA1* variants may not have been detected. With regard to ethnic backgrounds, these databases have similar ancestry groups (US study 94% self‐reported White, UK Biobank 93.5% non‐Finnish European ancestry) and are unlikely to be accountable for the discrepancies we have observed between studies.^14^

No female carriers were identified, in contrast to a US study that found a high proportion of heterozygous women with UBA1 mutations (VAF < 30%).^13^ The latter study included a greater proportion of African American (27%) and Latino (19%) individuals, populations underrepresented in the UK Biobank. None of these heterozygous female carriers exhibited clinical features of VEXAS or other inflammatory/myeloid conditions, suggesting low disease penetrance in this group.

Analysis of the 100 000 Genomes Project, which included over 85 000 participants, identified only one possible VEXAS syndrome case. The majority of participants were recruited for non‐haematological rare disorders, and the prevalence discrepancies with the US studies may also be due to a significant proportion of the 100kGP cohort being children. The sole case, a male over 50, was found in the 100kGP cancer programme within the MPN/MDS arm of the haematological malignancy cohort. MPN/MDS patients in this study were recruited only if they lacked known MDS‐defining mutations, as the study aimed to uncover novel genetic drivers of MDS. Given that many undiagnosed VEXAS cases were previously classified as MDS without genetic confirmation,^9^ it is unsurprising that the only VEXAS case emerged from this specific patient group.

We then proceeded to interrogate the data from several diagnostic laboratories. The UK's National Health Service provides a centralized genetic testing system for autoinflammatory conditions, allowing for a comprehensive assessment of VEXAS syndrome prevalence. Among individuals investigated for possible autoinflammatory disorders, the prevalence was nearly 1%, with 100% disease penetrance—every individual with a canonical *UBA1* mutation was diagnosed with VEXAS syndrome. This is in keeping with previous reports showing that male patients with the pathogenic *UBA1* mutations invariably exhibit disease manifestations in keeping with VEXAS syndrome.^4^, ^10^, ^15^ Only one female case was identified, reaffirming the male predominance of VEXAS.

The largest group of VEXAS syndrome patients was identified among those investigated for unexplained cytopenias and suspected myeloid malignancies. The prevalence was higher in the Leeds HMDS cohort than in the King's HMDS cohort, likely because Leeds was the first UK centre to offer routine *UBA1* genetic testing. Nearly half of diagnosed cases in Leeds were suspected of having VEXAS from the outset. The true prevalence of undiagnosed VEXAS among myeloid disorder patients is likely closer to estimates from the King's HMDS cohort, where most pre‐test diagnoses were MDS. However, prevalence figures from both cohorts suggest VEXAS syndrome is relatively common and predictable in this population. Unlike previous studies focusing solely on confirmed MDS cases,^9^ these cohorts provide real‐world insights by including all patients investigated for myeloid conditions.

These findings highlight the need to incorporate *UBA1* mutation analysis into diagnostic panels for unexplained cytopenia and suspected MDS. However, not all patients with canonical *UBA1* mutations were diagnosed with VEXAS syndrome, suggesting incomplete disease penetrance. Several factors may explain this. In some cases, clinical history was incomplete, and diagnoses relied on limited information from referring clinicians. Additionally, many patients were classified as having MDS despite lacking other defining mutations, raising debate over whether such cases should be reclassified as VEXAS, given their distinct bone marrow features and prognosis.^16^, ^17^ The absence of fully established diagnostic criteria for VEXAS syndrome further complicates classification. These findings underscore the need for revised diagnostic frameworks that account for the unique haematological manifestations of VEXAS, particularly in *UBA1*‐driven cases.

With *UBA1* now included in routine diagnostic panels for autoinflammatory and myeloid disorders, diagnostic laboratories increasingly report VUSs. Some of these have been confirmed as pathogenic, though non‐Met41 variants often present differently from classical VEXAS syndrome. These patients typically exhibit less inflammation, with bone marrow failure as the predominant feature.^7^, ^18^ However, this is not always accompanied by the typical MDS or VEXAS diagnostic features on bone marrow examination.^18^

Most VUSs remain uncharacterized. We investigated their potential pathogenicity by assessing whether they cluster within specific patient cohorts or functional domains of the *UBA1*‐encoded E1 enzyme. As an indirect measure, we compared their VAF and co‐occurrence with MDS‐causing mutations to canonical *UBA1* mutations.

We identified four unique VUSs in 100kGP, 20 in the Leeds HMDS cohort and 30 in the King's HMDS cohort, distinct from p.Met41 variants. While their pathogenicity remains uncertain, several indicators suggest a disease‐associated role. Most were found in men with myeloid malignancies and had VAFs comparable to canonical variants. Three were recurrent, one appearing in four cases, and several demonstrated clonal dominance over other MDS‐causing mutations. Structural analysis revealed that many VUSs, including recurrent ones, cluster in the adenylation domain, a region where other mutations have been shown to disrupt E1 ligase activity.^2^, ^7^ However, functional studies on VUSs will be essential to delineate their roles in pathogenesis. We did not include any variants with a possible germline origin in our analysis, as illustrated by SMA even variants that appear to have relatively moderate effects on UBA1 function cause early‐onset, severe multisystem disease, which we had not seen in the cohorts we studied.

In conclusion, our study shows that, in the general UK population, the prevalence of VEXAS syndrome is not as high as reported elsewhere. However, the condition is not rare, with a relatively high estimated incidence in selected populations undergoing investigations for myeloid disorders. Certain clinical features are highly suggestive of VEXAS syndrome, and some investigators have proposed scoring criteria to help identify candidates for genetic testing and improve diagnostic accuracy.^19^ However, larger studies are needed to validate this approach. Our findings advocate for comprehensive genetic testing in patients with unexplained cytopenias and suspected MDS, emphasizing the diagnostic importance of considering both canonical mutations and VUSs. Future research should focus on refining diagnostic criteria and elucidating the molecular mechanisms underlying VEXAS to improve patient care and outcomes.

## AUTHOR CONTRIBUTIONS

SS and CC conceived the study. AMR, LC, JAP and AS analysed the data. SS, CC, DR, AM, SF, AA, RT, KKL, HL and AK contributed data and validated clinical information. AMR, LC and SS wrote the first draft of the manuscript. SS provided funding. All authors read, edited and approved the manuscript.

## FUNDING INFORMATION

SS is funded by Kennedy Trust Senior Fellowship and MRC project grant number MR/Y011945/1. AA, AS, CC and SS are supported in part by the National Institute for Health and Care Research (NIHR) Leeds Biomedical Research Centre (BRC) (NIHR203331). The views expressed are those of the author(s) and not necessarily those of the NHS, the NIHR or the Department of Health and Social Care. JAP is funded by a UKRI Future Leaders Fellowship (MR/Y034325/1).

## CONFLICT OF INTEREST STATEMENT

The authors declare no conflicts of interest.

## ETHICS APPROVAL STATEMENT

This research has been conducted using data from UK Biobank, a major biomedical database. UK Biobank website: www.ukbiobank.ac.uk. This research has been conducted under Application Number 82060. All data analysed from Genomics England were conducted under the project registry ID 1183. Research on the de‐identified patient data used in this publication can be carried out in the Genomics England Research Environment subject to a collaborative agreement that adheres to patient‐led governance. All interested readers will be able to access the data in the same manner that the authors accessed the data. For more information about accessing the data, interested readers may contact research-network@genomicsengland.co.uk or access the relevant information on the Genomics England website: https://www.genomicsengland.co.uk/research. This research was also made possible through access to data in the National Genomic Research Library, which is managed by Genomics England Limited (a wholly owned company of the Department of Health and Social Care). The National Genomic Research Library holds data provided by patients and collected by the NHS as part of their care and data collected as part of their participation in research. The National Genomic Research Library is funded by the National Institute for Health Research and NHS England. The Wellcome Trust, Cancer Research UK and the Medical Research Council have also funded research infrastructure.

## Supporting information


Data S1.

## Data Availability

The data that support the findings of this study are available from the corresponding author upon reasonable request.
