# Supplementary material for: Mapping VEXAS‐associated and rare UBA1 variants in the United Kingdom: Insights from patient cohorts and the general population
Source: Br J Haematol. 2025 May 25;208(1):116–28. doi: 10.1111/bjh.20176 (PMC12819087; doi:10.1111/bjh.20176)
Supplement: Supplementary file 1 — Data S1. [file BJH-208-116-s001.zip › bjh20176-sup-0001-Supplementary S1.docx]

**Supplementary methods**

UK Biobank

The UK Biobank is a vast online data repository that aims to enable scientific discoveries and improve human health^1^. The initiative has gathered data from 500,000 participants aged 40-69 (roughly in an even male-female ratio) recruited between 2006 and 2010 throughout the UK, with participants attending one of 22 assessment centres located across England, Scotland, and Wales. The information includes biochemistry and primary care records, imaging, cognitive and electrocardiogram metrics, and details on lifestyle and environmental factors. Genomic data (genome-wide genotype data, whole genome and whole exome sequencing) are available on request and can be used with main bioinformatic tools. This analysis was conducted under application number 82060.

The UK Biobank exome data was obtained using the Original Quality Functional Equivalent (OQFE) protocol as detailed previously^2-4^. In summary, all the sequencing files were mapped using BWA MEM to the GRCh38 reference genome and small variants were identified using WeCall^2^ . Single nucleotide variant (SNV) genotypes with read depth less than 7× and indel genotypes with read depth less than 10× were assigned as no-call genotypes^2^. All *UBA1* variants from the GLnexus joint-genotyped, multi-sample project-level variant call format files (pVCFs), generated from 469,589 gVCF files, were pulled out and annotated using the Variant Effect Predictor (VEP) version 113^5^. Variants on the canonical *UBA1* transcript were filtered by population frequency (MAF < 1%), CADD score (>20) and functional consequences (frameshift, inframe deletion, missense, splice donor variants, and stop gained)^6^. Additionally, we included further pathogenicity prediction annotation using AlphaMissense (AM) and Rare Exome Variant Ensemble Learner (REVEL) scores.^7,8^Due to the cost and computational requirements for running a bioinformatics pipeline using the whole cohort, we calculated the average sequencing depth of all *UBA1* exons with a small collection of randomly selected samples (498 females and 485 males) using samtools coverage^9^ (Supplementary Table 2).

100,000 Genomes Project

The Genomic England 100,000 Genomes Project (100kGP) was a UK initiative aiming to sequence 100,000 whole genomes in over 85,000 participants recruited for either rare diseases or cancer^10^. Data release v19 of 100kGP contains genomes from 72,884 rare disease participants and 15,621 participants of the cancer programme, along with their clinical and phenotype data (<https://re-docs.genomicsengland.co.uk/release19/>). These participants cover a wide age range (years of birth between 1917-2020) and of approximately even male-female ratio. All analysis was conducted through the Genomics England Research Environment under the project registry ID 1183.

The somatic tumor samples in the cancer programme were interrogated using the somAgg v0.2 workflow (<https://re-docs.genomicsengland.co.uk/somAgg/>), containing a total of 16,341 sequenced samples aligned to human reference genome GRCh38 and analysed by Strelka v2.4.7. We focused our analysis on genomes from blood samples only (842 samples). The somAgg workflow was created using an older version of 100kGP (version 12) and participants who have since withdrawn from the programme have been excluded from our results. Tumor samples have an average coverage of 100× with matched germline samples at 30× coverage. Variant genotypes and functional annotations were extracted from the aggregate somatic VCFs using bcftools v1.6^11^. Sample phenotypes were extracted from LabKey API (main programme v19) using R. The prevalence of *UBA1* variants in germline samples (average 30× coverage) across both rare disease and cancer programmes were investigated using the 100kGP small variant workflow v3.1.2. A total of 77,195 participants (62,729 rare disease; 14,466 cancer) from 100kGP (version 19) were investigated in the workflow after sub-setting for sample type (blood only), genome build (GRCh38), and delivery version (Illumina V4). All variants were then filtered by the canonical *UBA1* transcript and the same parameters described for the UK Biobank dataset.

Autoinflammatory gene panel and testing

The autoinflammatory panel includes the following genes: *IL1RN, IL36RN, LPIN2, MEFV, MVK, NLRC4, NLRP12, NLRP3, NOD2, OTULIN, PLCG2, PSMB4, PSMB8, PSMB9, PSTPIP1, RBCK1, SH3BP2, SLC29A3, TNFRSF1A, TMEM173, TNFAIP3, UBA1, CARD14, ADA2* (also known as *CECR1*). The libraries were prepared using Illumina AmpliSeq custom kits, covering coding regions and flanking intron sequences of the above-listed genes. Sequencing reactions were performed on Illumina MiSeq sequencer with the minimum amplicon coverage of ≥200×, DNA results were aligned to the reference human genome GRCh37. Genomic data was filtered and analysed using Illumina Base Space Variant Interpreter software (https://variantinterpreter.informatics.illumina.com/home).

Leeds HMDS sequencing method

The Leeds HMDS gene panel includes haematological malignancies associated genes (Supplementary Table 1). For the preparation of libraries, the laboratory uses Twist Bioscience reagents and samples are sequenced in an IIllumina NextSeq 550 platform. Reads are aligned to reference genome GRCh37p13. The minimum reporting thresholds are 5% VAF (variant allele frequency) and 100× read depth at the identified genomic position. Thresholds for ARID1A and KMT2C are set at 20% VAF due to higher frequencies of technical artefacts for these genes.

King’s College Hospital HMDS sequencing method

The King’s College HMDS gene panel includes haematological malignancies associated genes (Supplementary Table 1). The sequencing method uses QiaSeq targeted amplicon and an Illumina NextSeq 550 platform. Reads are aligned to reference genome GRCh37. For regions with read depth >400x, the limit of detection is 5%. In regions with sub-optimal coverage, variant may be missed. These are listed below by gene name and exon. Known variants below 5% frequency may be reported if clinically relevant. Novel variants below 5% are not reported.

Statistics

95% confidence intervals of prevalence values were calculated using the Poisson distribution and the exact-c method using the statmodels package in Python.

Reference:

1 Biobank, U. *About our data*, <<https://www.ukbiobank.ac.uk/enable-your-research/about-our-data>> (

2 Backman, J. D. *et al.* Exome sequencing and analysis of 454,787 UK Biobank participants. *Nature* **599**, 628-634 (2021). <https://doi.org/10.1038/s41586-021-04103-z>

3 Szustakowski, J. D. *et al.* Advancing human genetics research and drug discovery through exome sequencing of the UK Biobank. *Nat Genet* **53**, 942-948 (2021). <https://doi.org/10.1038/s41588-021-00885-0>

4 Biobank, U. *Final Exome Data Release FAQs / July 2022*, <<https://www.ukbiobank.ac.uk/media/najcnoaz/access_064-uk-biobank-exome-release-faq_v11-1_final-002.pdf>). > (

5 McLaren, W. *et al.* The Ensembl Variant Effect Predictor. *Genome Biol* **17**, 122 (2016). <https://doi.org/10.1186/s13059-016-0974-4>

6 Schubach, M., Maass, T., Nazaretyan, L., Roner, S. & Kircher, M. CADD v1.7: using protein language models, regulatory CNNs and other nucleotide-level scores to improve genome-wide variant predictions. *Nucleic Acids Res* **52**, D1143-D1154 (2024). <https://doi.org/10.1093/nar/gkad989>

7 Cheng, J. *et al.* Accurate proteome-wide missense variant effect prediction with AlphaMissense. *Science* **381**, eadg7492 (2023). <https://doi.org/10.1126/science.adg7492>

8 Ioannidis, N. M. *et al.* REVEL: An Ensemble Method for Predicting the Pathogenicity of Rare Missense Variants. *Am J Hum Genet* **99**, 877-885 (2016). <https://doi.org/10.1016/j.ajhg.2016.08.016>

9 Danecek, P. *et al.* Twelve years of SAMtools and BCFtools. *Gigascience* **10** (2021). <https://doi.org/10.1093/gigascience/giab008>

10 Caulfield, M. D., Jim; Dennys, Martin; Elbahy, Leila; Fowler, Tom; Hill, Sue; et al. (2017).

11 Li, H. A statistical framework for SNP calling, mutation discovery, association mapping and population genetical parameter estimation from sequencing data. *Bioinformatics* **27**, 2987-2993 (2011). <https://doi.org/10.1093/bioinformatics/btr509>
