# Supplementary material for: Mapping VEXAS‐associated and rare UBA1 variants in the United Kingdom: Insights from patient cohorts and the general population
Source: Br J Haematol. 2025 May 25;208(1):116–28. doi: 10.1111/bjh.20176 (PMC12819087; doi:10.1111/bjh.20176)
Supplement: Supplementary file 1 — Data S1. [file BJH-208-116-s001.zip › bjh20176-sup-0002-Supplementary Tables1-4.docx]

| Leeds HMDS gene panel | | | | | |
| --- | --- | --- | --- | --- | --- |
| Gene | **Reference (NM)** | **Gene2** | **Reference (NM)2** | **Gene3** | **Reference (NM)3** |
| ANKRD26 | NM_014915.2 | **GNB1** | NM_002074.4 | **PRPF8** | NM_006445.3 |
| ASXL1 | NM_015338.5 | **HRAS** | NM_005343.2 | **PTEN** | NM_000314.6 |
| BCOR | NM_001123385.1 | **IDH1** | NM_005896.3 | **PTPN11** | NM_002834.4 |
| BCORL1 | NM_001184772.2 | **IDH2** | NM_002168.3 | **RAD21** | NM_006265.2 |
| CALR | NM_004343.3 | **IKZF1** | NM_006060.5 | **RUNX1** | NM_001754.4 |
| CBL | NM_005188.2 | **JAK2** | NM_004972.3 | **SETBP1** | NM_015559.2 |
| CEBPA | NM_004364.4 | **KIT** | NM_000222.2 | **SF3B1** | NM_012433.3 |
| CSF3R | NM_156039.3 | **KMT2A*** | PTD | **SH2B3** | NM_005475.2 |
| CUX1 | NM_001913.4 | **KMT2C** | NM_170606.2 | **SRSF2** | NM_003016.4 |
| DDX41 | NM_016222.3 | **KRAS** | NM_004985.4 | **STAG2** | NM_001042749.2 |
| DNMT3A | NM_175629.2 | **MPL** | NM_005373.2 | **STAT3** | NM_139276.2 |
| ETNK1 | NM_016838.4 | **NF1** | NM_001042492.2 | **STAT5B** | NM_012448.3 |
| ETV6 | NM_001987.4 | **NFE2** | NM_001261461.1 | **TET2** | NM_001127208.2 |
| EZH2 | NM_004456.4 | **NOTCH1** | NM_017617.4 | **TP53** | NM_000546.5 |
| FBXW7 | NM_033632.3 | **NPM1** | NM_002520.6 | **U2AF1** | NM_006758.2 |
| FLT3 | NM_004119.2 | **NRAS** | NM_002524.4 | **UBA1** | NM_033343.3 |
| GATA1 | NM_002049.3 | **PHF6** | NM_001158771.1 | **WT1** | NM_024424.4 |
| GATA2 | NM_001145661.1 | **PPM1D** | NM_003620.3 | **ZRSR2** | NM_005089.3 |
| King’s College HMDS gene panel | | | | | |
| Gene | **Reference (NM)** | **Gene2** | **Reference (NM)2** | **Gene3** | **Reference (NM)3** |
| ANKRD26 | NM_014915.3 | **GNB1** | NM_002074.5 | **PPM1D** | NM_003620.4 |
| ASXL1 | NM_015338.6 | **HRAS** | NM_005343.4 | **PTPN11** | NM_002834.5 |
| BCL2 | NM_000633.3 | **IDH1** | NM_005896.4 | **RAD21** | NM_006265.3 |
| BCOR | NM_001123385.2 | **IDH2** | NM_002168.4 | **RUNX1** | NM_001754.5 |
| CALR | NM_004343.4 | **IKZF1** | NM_006060.6 | **SETBP1** | NM_015559.3 |
| CBL | NM_005188.4 | **JAK2** | NM_004972.4 | **SF3B1** | NM_012433.4 |
| CEBPA | NM_004364.5 | **KIT** | NM_000222.3 | **SH2B3** | NM_005475.3 |
| CSF3R | NM_156039.3 | **KMT2A** | NM_001197104.2 | **SRSF2** | NM_003016.4 |
| CUX1 | NM_181552.4 | **KMT2C** | NM_170606.3 | **STAG2** | NM_001042749.2 |
| DDX41 | NM_016222.4 | **KRAS** | NM_033360.4 | **STAT5B** | NM_012448.4 |
| DNMT3A | NM_022552.5 | **MPL** | NM_005373.3 | **TET2** | NM_001127208.2 |
| ETV6 | NM_001987.5 | **NF1** | NM_001042492.3 | **TP53** | NM_000546.6 |
| EZH2 | NM_004456.5 | **NFE2** | NM_001136023.3 | **U2AF1** | NM_006758.3 |
| FLT3 | NM_004119.3 | **NPM1** | NM_002520.6 | **UBA1** | NM_003334.4 |
| GATA1 | NM_002049.4 | **NRAS** | NM_002524.5 | **WT1** | NM_024426.6 |
| GATA2 | NM_032638.5 | **PHF6** | NM_032458.3 | **ZRSR2** | NM_005089.4 |

**Supplementary Table 1: Leeds & King’s College Haematological Malignancy Diagnostic Service (HMDS) Virtual Gene Panel (acute and myeloid).**

| Dataset | BAM files assessed | Mean depth across UBA1 | Sensitivity/Limit of detection (LOD) |
| --- | --- | --- | --- |
| 100kGP | Cancer cohort (n=842) | 58× in males  109× in females | Estimated sensitivity 0.91 for SNVs and 0.90 for indels at 100× |
|  | Rare disease (n=77,000) | 20× in males  40× in females | Estimated sensitivity 0.999 for SNVs and 0.936 for indels at 100× |
| UK Biobank | n=983* | 38× in males  74× in females | Estimation LOD 18%  Estimation LOD 9% |
| Leeds HMDS | Overall screening | 158× in males  296× in females | LOD 5% |
| King’s College HMDS | n=35 | 2279× in males  3889× in females | LOD 5% |
| Autoinflammatory diagnostic cohort | n=20 | 641× in males  799× in females | LOD 3% |

**Supplementary Table 2: UBA1 sequencing information across cohorts.** Table includes the number of BAM files analysed for coverage metrics. The mean sequencing depth across *UBA1* is displayed for male and female participants. Limits of detection (LOD) for 100kGP are not available and their reported sensitivity has been supplied instead; LOD for UKB are estimated based on average coverage and the minimum read depth called being 7 reads. *983 BAM files were randomly selected from UK Biobank to estimate the average coverage of the gene.

| **Sex** | **Age** | **Gene** | **gDNA Nomenclature** | **CDS Nomenclature** | **Protein**  **Nomenclature** | **RefSeq transcript** | **VAF** | **Consequence** | **COSMIC** | **SIFT Description** | **PolyPhen Description** | **CADD score** | **AM score** | **Inflammatory**  **conditions** |
| --- | --- | --- | --- | --- | --- | --- | --- | --- | --- | --- | --- | --- | --- | --- |
| M | >50 | *UBA1* | ChrX:g.47210107 A>G | c.2183G>A | p.(Asn728Ser) | NM_003334.4 | 39% | Missense | N/A | Deleterious | - | 23.3 | 0.134 (LB) | No |
| M | >50 | *UBA1* | ChrX:g.47212804 G>A | c.2587A>G | p.(Val863Met) | NM_003334.4 | 54% | Missense | N/A | Deleterious | - | 24.7 | 0.455 (A) | Yes |

**Supplementary Table 3: Putative somatic variants in *UBA1* identified in UK Biobank (males only).** Genomic coordinates are provided for the human reference genome build GRCh38. CDS nomenclature provided according to the assigned RefSeq transcript. AlphaMissense (AM) scores classified as likely benign (LB), ambiguous (A) and likely pathogenic (LP).

| **Sex** | **Age** | **Gene** | **gDNA Nomenclature** | **CDS**  **Nomenclature** | **Protein**  **Nomenclature** | **RefSeq transcript** | **VAF** | **Consequence** | **COSMIC** | **SIFT Description** | **Polyphen Description** | **CADD**  **score** | **AM score** | **Disease sub type** |
| --- | --- | --- | --- | --- | --- | --- | --- | --- | --- | --- | --- | --- | --- | --- |
| F | >50 | *UBA1* | ChrX:g.47201326G>T | c.638G>T | p.(Gly213Val) | NM_003334.4 | 2.8% | Missense | N/A | Deleterious | Probably damaging | 25.1 | 0.989 (LP) | Acute myeloid leukaemia |
| M | <50 | *UBA1* | ChrX:g.47214834G>T | c.3082G>T | p.(Gly1028Cys) | NM_003334.4 | 5.4% | Missense | COSV60114532 | Deleterious | Possibly damaging | 26.4 | 0.402 (A) | Acute myeloid leukaemia |
| M | <50 | *UBA1* | ChrX:g.47202994C>A | c.1285C>A | p.(Leu429Ile) | NM_003334.4 | 8.2% | Missense | COSV100255686 | Tolerated | Benign | 20.6 | 0.113 (LB) | Acute myeloid leukaemia |
| M | <50 | *UBA1* | ChrX:g.47201485C>A | c.686C>A | p.(Pro229His) | NM_003334.4 | 5.9% | Missense | N/A | Deleterious | Benign | 21.5 | 0.268 (LB) | Acute myeloid leukaemia |
| M | >50 | *UBA1* | ChrX:g.47214897G>A | c.3145G>A | p.(Glu1049Lys) | NM_003334.4 | 75.7% | Missense | N/A | Deleterious | Probably damaging | 24.4 | 0.979 (LP) | Acute myeloid leukaemia |

**Supplementary Table 4: Putative somatic variants in *UBA1* identified in the GEL cancer programme, from participants with haematological oncology (HAEMONC).** Genomic coordinates are provided for the human reference genome build GRCh38. CDS nomenclature provided according to the assigned RefSeq transcript. AlphaMissense (AM) scores classified as likely benign (LB), ambiguous (A) and likely pathogenic (LP).
