# Supplementary figures and images for: Mapping VEXAS‐associated and rare UBA1 variants in the United Kingdom: Insights from patient cohorts and the general population
Source: Br J Haematol. 2025 May 25;208(1):116–28. doi: 10.1111/bjh.20176 (PMC12819087; doi:10.1111/bjh.20176)

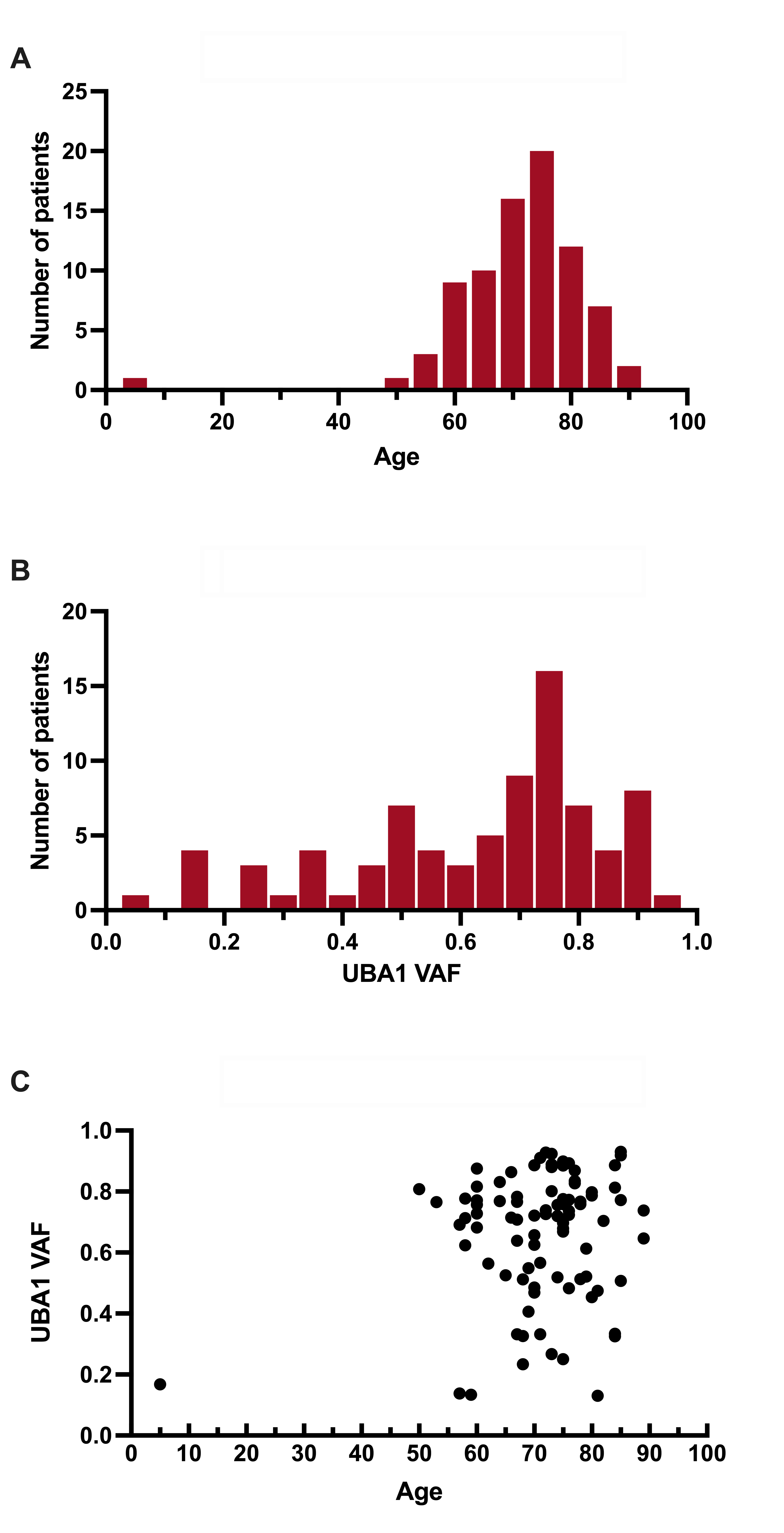

Supplement: Supplementary file 1 — Data S1. [file BJH-208-116-s001.zip › bjh20176-sup-0004-FigS2.tiff]

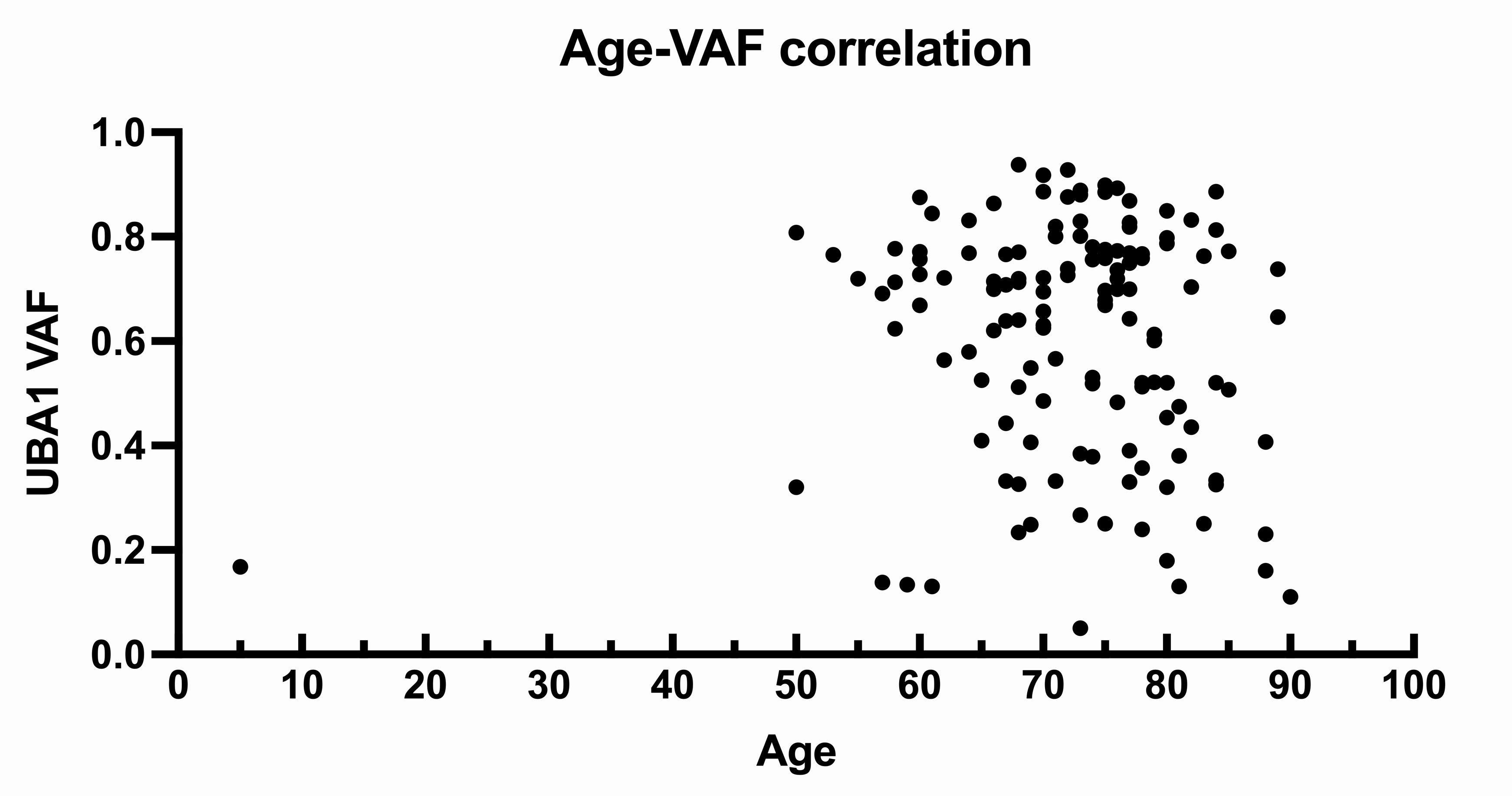

Supplement: Supplementary file 1 — Data S1. [file BJH-208-116-s001.zip › bjh20176-sup-0005-FigS3.tiff]
